# Wfs1- deficient rats develop primary symptoms of Wolfram syndrome: insulin-dependent diabetes, optic nerve atrophy and medullary degeneration

**DOI:** 10.1038/s41598-017-09392-x

**Published:** 2017-08-31

**Authors:** Mario Plaas, Kadri Seppa, Riin Reimets, Toomas Jagomäe, Maarja Toots, Tuuliki Koppel, Tuuli Vallisoo, Mait Nigul, Indrek Heinla, Riho Meier, Allen Kaasik, Andres Piirsoo, Miriam A. Hickey, Anton Terasmaa, Eero Vasar

**Affiliations:** 1 Institute of Biomedicine and Translational Medicine, Department of Physiology, University of Tartu, 19 Ravila Street, Tartu, 50411 Estonia; 2 Institute of Biomedicine and Translational Medicine, Laboratory Animal Centre, University of Tartu, 14B Ravila Street, Tartu, 50411 Estonia; 3 Institute of Molecular and Cell Biology, University of Tartu, 23 Riia Street, Tartu, 51010 Estonia; 4 Institute of Biomedicine and Translational Medicine, Department of Pharmacology, University of Tartu, 19 Ravila Street, Tartu, 50411 Estonia; 5 Institute of Biomedicine and Translational Medicine, Department of Biomedicine, University of Tartu, 19 Ravila Street, Tartu, 50411 Estonia; 6 Centre of Excellence for Genomics and Translational Medicine, University of Tartu, Ravila 19, Tartu, 50411 Estonia

## Abstract

Wolfram syndrome (WS) is a rare autosomal-recessive disorder that is caused by mutations in the *WFS1* gene and is characterized by juvenile-onset diabetes, optic atrophy, hearing loss and a number of other complications. Here, we describe the creation and phenotype of *Wfs1* mutant rats, in which exon 5 of the *Wfs1* gene is deleted, resulting in a loss of 27 amino acids from the WFS1 protein sequence. These Wfs1-ex5-KO232 rats show progressive glucose intolerance, which culminates in the development of diabetes mellitus, glycosuria, hyperglycaemia and severe body weight loss by 12 months of age. Beta cell mass is reduced in older mutant rats, which is accompanied by decreased glucose-stimulated insulin secretion from 3 months of age. Medullary volume is decreased in older Wfs1-ex5-KO232 rats, with the largest decreases at the level of the inferior olive. Finally, older Wfs1-ex5-KO232 rats show retinal gliosis and optic nerve atrophy at 15 months of age. Electron microscopy revealed axonal degeneration and disorganization of the myelin in the optic nerves of older Wfs1-ex5-KO232 rats. The phenotype of Wfs1-ex5-KO232 rats indicates that they have the core symptoms of WS. Therefore, we present a novel rat model of WS.

## Introduction

Wolfram syndrome (WS) was first described in 1938 by Wolfram and Wagener as juvenile-onset diabetes with optic nerve atrophy^1^. The disorder is also named DIDMOAD (diabetes insipidus, diabetes mellitus, optic atrophy and deafness). WS is an autosomal-recessive disease caused by mutations in the *WFS1* gene^2^, which consists of 8 exons that encode a protein of 890 amino acids (aa) (approximately 100 kDa) called Wolframin^3, 4^. This protein is localized at the endoplasmic reticulum (ER) membrane^5^ but is also found in the secretory granules of pancreatic beta cells^6^. Despite extensive effort by many research groups, the precise molecular functions of the WFS1 protein are still unknown. However, a deficit of properly functioning Wolframin leads to impaired insulin secretion, premature beta cell death, and degeneration of optic nerve and other neurons in the central nervous system, collectively causing WS^7^. A wide spectrum of mutations are known in the *WFS1* gene; many, but not all lead to WS^3, 8^. Currently, there is no cure for WS, and treatment is limited to the management of symptoms^7^. Therefore, the development of animal models of WS is important for evaluating and developing necessary treatment strategies.

Several mouse models with disrupted Wfs1 function have been developed. Disruption of exon 2 of the *Wfs1* gene resulted in mice that showed impaired glucose-stimulated insulin secretion and increased rates of beta cell death^9^. Mice with beta cell-specific disruption of Wfs1 also show impaired glucose-induced insulin secretion and progressive glucose intolerance^10^. In our laboratory, whole-body Wfs1 mutant mice were created by replacing exon 8 of the *Wfs1* gene with an in-frame NLSLacZNeo cassette, resulting in a deletion corresponding to aa 360–890 of the WFS1 protein^11^. These Wfs1 mutant mice also show impaired glucose tolerance and lower insulin levels, and they have significantly lower body weights than wild-type (WT) littermates^12, 13^.

The different Wfs1 mutant mice that have currently been created show glucose intolerance and diminished insulin secretion. However, to the best of our knowledge, no mutant Wfs1 mice display fasting hyperglycaemia. All previous models of WS were created in mice, and they seem to only partially develop diabetes and other symptoms of WS; therefore, the aim of this study was to produce a new model of WS in a different species that could better mimic the human condition. Thus, we created and characterized a new transgenic rat model of WS.

## Results

### Exon 5 of the Wfs1 gene is deleted in Wfs1-ex5-KO232 rats

Three different Wfs1 mutant rat lines were created using zinc-finger technology. Based on genomic sequences, two of the lines (Wfs1-ex5-KO232 and Wfs1-ex5-KO266) had a deletion of exon 5 of the *Wfs1* gene, and the third line (Wfs1-ex5-INS244) had a substitution in exon 5 of the *Wfs1* gene, which is predicted to result in a substitution of LQK (aa 224–226) into YCMNTI in the WFS1 protein (Supplementary Figure S1). Rats from every line showed glucose intolerance at 7 months of age (Supplementary Figure S2). Only the Wfs1-ex5-KO232 line was characterized and used (males only) in the subsequent experiments. All 3 Wfs1 mutant lines are currently being maintained for future studies.

**Figure 1:**
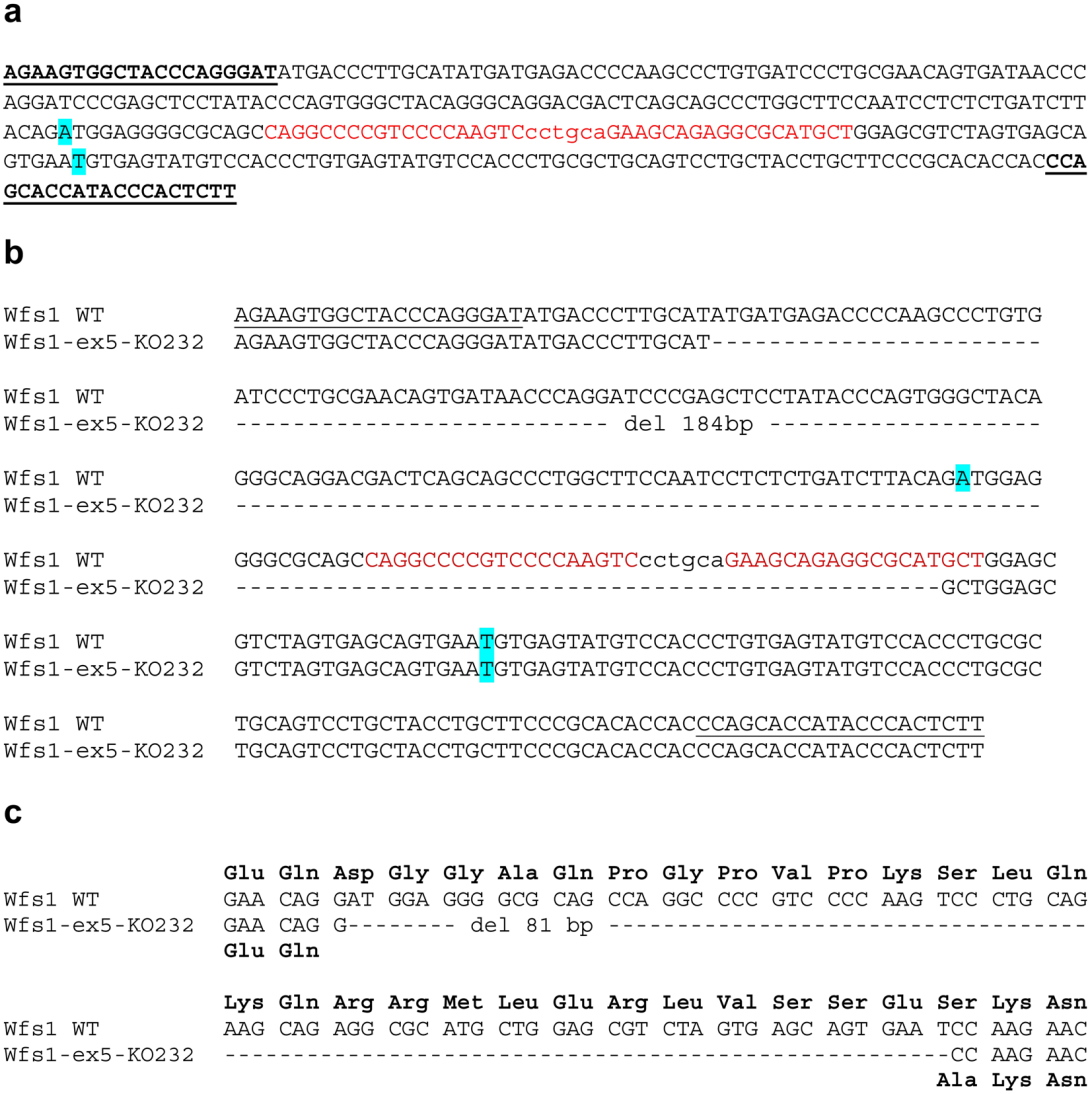
Creation and characterization of the Wfs1 mutation in rats. **(a)** Zink finger nuclease (ZFN) design and cutting site. Genotyping primers are in bold and underlined, ZFN binding site in red, ZFN cut site in lower case red, and blue indicates the start and end of exon 5 of the rat *Wfs1* gene. (**b)** DNA Sequence of exon 5 of the Wfs1 deficient rat line 232. Rat line Wfs1-ex5-KO232 lost 184 bp (17,833–18,017) in the *Wfs1* gene, including 55 bp in exon 5; blue indicates the start and end of exon 5. **(c)** Comparison of cDNA and protein sequences from wild-type (WT) and Wfs1-ex5-KO232 rat lines. Line Wfs1-ex5-KO232 has lost all nucleotides from coding exon 5 of the *Wfs1* gene. According to cDNA sequencing and ORF analysis, the resulting strain had lost exon 5 in the *Wfs1* gene. Protein sequence is predicted from cDNA analysis; deletion of 55 bp from exon 5 of the rat *Wfs1* gene did not result in a frame shift mutation. Thus, there is a loss of 27 amino acids (from coding exon 5 of the *Wfs1* gene) and a new GCC codon (coding A - alanine) at the junction of exon 4 and exon 6 in the Wfs1-ex5-KO232 rats (marked red).

Mutation of Wfs1 in Wfs1-ex5-KO232 rats was verified by pyrosequencing cDNA, which confirmed a deletion of exon 5 of the rat *Wfs1* gene (Fig. 1c). This deletion is predicted to result in a loss of 27 aa from the WFS1 protein sequence (aa 212–238) and a substitution of serine to alanine at position 239 (Fig. 1c) in Wfs1-ex5-KO232 rats.

### Wfs1-ex5-KO232 rats develop glucose intolerance and diabetes mellitus

**Figure 2:**
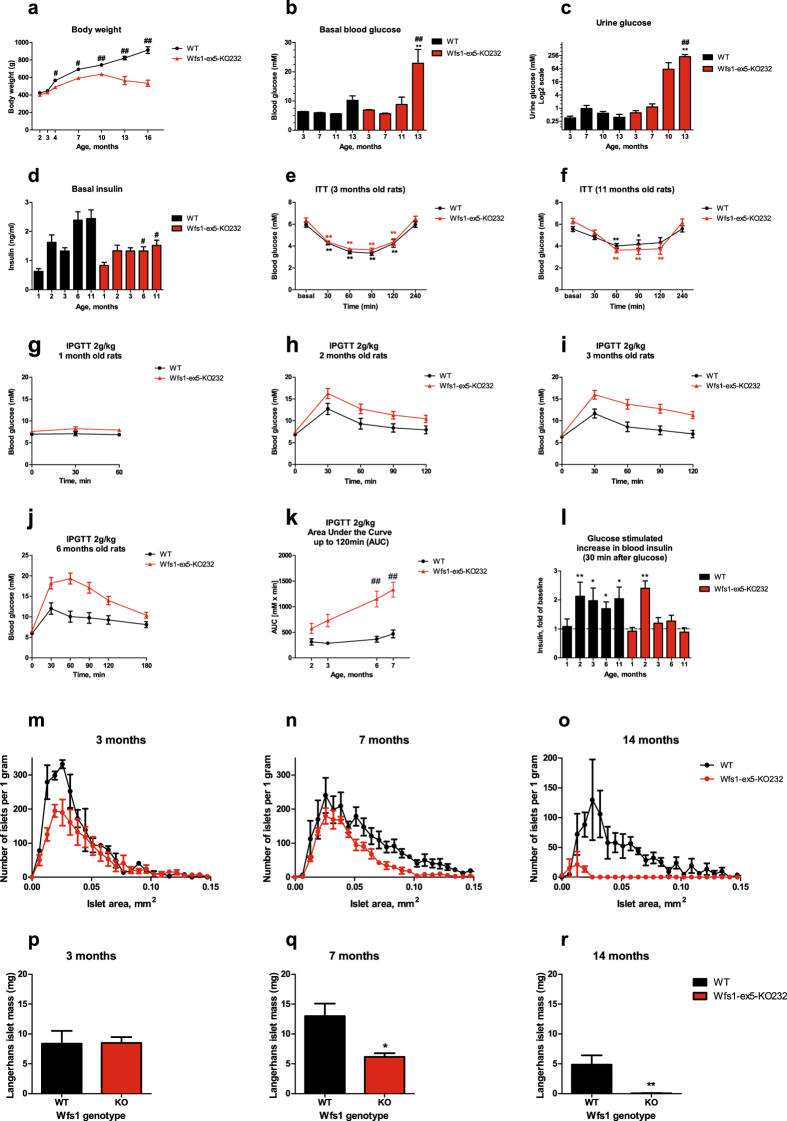
Development of diabetes mellitus in Wfs1-ex5-KO232 rats. **(a)** Wfs1-ex5-KO232 animals are slightly lighter than wild-type (WT) rats of similar ages and begin to lose weight after 10 months of age. **(b)** Basal blood glucose levels were similar for both genotypes up to 11 months of age; thereafter, Wfs1-ex5-KO232 rats develop hyperglycaemia. **(c)** Wfs1-ex5-KO232 rats developed glycosuria after 10 months of age. **(d)** Insulin levels were lower in older Wfs1-ex5-KO232 animals compared to levels in WT animals of the same age. For the insulin tolerance tests (ITTs), human insulin was administered (1 U/kg, s.c.), and blood glucose levels were measured at the indicated time points. There were no genotype-associated changes in insulin sensitivity in either **(e)** young or **(f)** old animals. For intraperitoneal glucose tolerance tests (IPGTTs), blood glucose levels were measured after administration of glucose (2 g/kg i.p.). **(g**,**h)** Glucose tolerance was similar for both genotypes at 1 and 2 months. **(i)** At 3 months of age, Wfs1-ex5-KO232 rats showed a slight glucose intolerance compared with WT rats, which was exacerbated at (**j**) 6 months. **(k)** Area under the curve for IPGTT results at different ages. **(l**) Glucose-stimulated increases in blood insulin levels (relative to baseline) 30 minutes after glucose administration, Wfs1-ex5-KO232 animals showed a defect in insulin secretion after 3 months of age. Size distribution of islets of Langerhans in rats at **(m)** 3, **(n)** 7 and **(o)** 14 months of age. Islet mass in rats at **(p)** 3, **(q)** 7 and **(r)** 14 months of age. The data were compared using two-way ANOVAs followed by Tukey’s HSD tests; ^#^p < 0.05, ^##^ < 0.01 between genotypes, *p < 0.05, **p < 0.01 within genotype (vs baseline and vs 3 months age). The data are presented as the mean ± SEM, n = 6–8.

Wfs1-ex5-KO232 rats were lighter than WT littermates 4–10 months of age. From 10 months, Wfs1-ex5-KO232 animals began to lose weight, whereas WT littermates continued to grow (Fig. 2a), (F(1,153) = 197.9, p < 0.001 (genotype); F(7, 153) = 114.5, p < 0.001 (age); F(7, 153) = 22.07, p < 0.001 (genotype × age)).

Blood glucose levels were indistinguishable between genotypes up to 11 months of age (Fig. 2b); thereafter, blood glucose levels in Wfs1-ex5-KO232 rats showed a dramatic increase and were greater than 20 mM at 13 months (Fig. 2b), (F(1,48) = 8.19, p = 0.006 (genotype); F(3, 48) = 12.88, p < 0.001 (age); F(3, 48) = 4.48, p < 0.007 (genotype × age)).

Urine glucose levels were similar for both genotypes up to 7 months of age. However, by 10 months, Wfs1-ex5-KO232 rats showed signs of glycosuria, which was further exacerbated at 13 months (Fig. 2c), (F(1,51) = 9.87, p = 0.003 (genotype); F(3, 51) = 5.34, p = 0.003 (age); F(3, 51) = 5.36, p = 0.003 (genotype × age)). Thus, glycosuria appears to precede the development of hyperglycaemia in Wfs1-ex5-KO232 rats.

Basal serum insulin levels in Wfs1-ex5-KO232 rats were lower than in WT littermates beginning at 6 months of age (Fig. 2d), (F(1,55) = 9.46, p = 0.003 (genotype); F(4,55) = 11.42, p < 0.001 (age); F(4, 55) = 3.53, p = 0.01 (genotype × age)).

Administration of human insulin reduced blood glucose levels in three-month-old (F(5,55) = 82.73, p < 0.001 (treatment)) and 11 months old rats (F(5,55) = 25.47, p < 0.001 (treatment)) (Fig. 2e,f). However, there was no difference in insulin sensitivity between Wfs1-ex5-KO232 and WT rats, regardless of age (Fig. 2e,f), (F(1,55) = 2.77, p = 0.12 (genotype, 3-month-old); (F(1,55) = 0.03, p = 0.86 (genotype, 11 months old)).

Wfs1-ex5-KO232 rats showed a tendency towards reduced glucose tolerance by 2–3 months of age (Fig. 2h and i). By 6 months of age, Wfs1-ex5-KO232 rats displayed significant glucose intolerance (Fig. 2j). An area-under-the-curve analysis of the glucose tolerance data revealed that glucose intolerance progressed steadily with age in Wfs1-ex5-KO232 rats, whereas there was no age-dependent change in glucose tolerance in WT littermate controls (Fig. 2k), (F(1,44) = 64.78, p < 0.001 (genotype); F(3,44) = 8.60, p < 0.001 (age); F(3, 44) = 3.75, p = 0.017 (genotype × age)).

Glucose administration induced an increase in blood insulin levels at 30 min post administration in WT rats at 2–11 months (Fig. 2l), whereas Wfs1-ex5-KO232 rats showed this increase only at 2 months (Fig. 2l), indicating a defect in glucose-stimulated insulin release in Wfs1-ex5-KO232 rats starting from 3 months of age (Fig. 2l). For unknown reasons, rats of both genotypes did not show an increase in blood glucose during glucose tolerance tests at 1 month of age (Fig. 2g).

### Beta cell mass is reduced in olderWfs1-ex5-KO232 rats

In accordance with the data from the glucose tolerance tests, qualitative and quantitative histological analyses of pancreases revealed age- and genotype-dependent alterations in islet mass and size (Fig. 2m–r). At three months of age, islet mass was similar in Wfs1-ex5-KO232 and WT littermates (Fig. 2p); at seven months, islet mass was reduced in Wfs1-ex5-KO232 rats (Fig. 2q), and islets had virtually disappeared in 14-month-old mutant rats (Fig. 2r). A size analysis of islets of Langerhans show similar size distributions in 3-month-old rats of both groups, although WT rats displayed a higher number of smaller islets (Fig. 2m), which apparently does not alter islet mass at this age (Fig. 2p). As animals grew older, there was an increase in the proportion of larger islets in WT animals; this was not observed in Wfs1-ex5-KO232 rats (Fig. 2n). Finally, in 14-month-old Wfs1-ex5-KO232 rats, only a small number of small islets were identified (Fig. 2o). 2–5 animals per genotype and age group were analysed for islet size.

### ER stress was detected in pancreases of Wfs1-ex5-KO232 rats

**Figure 3:**
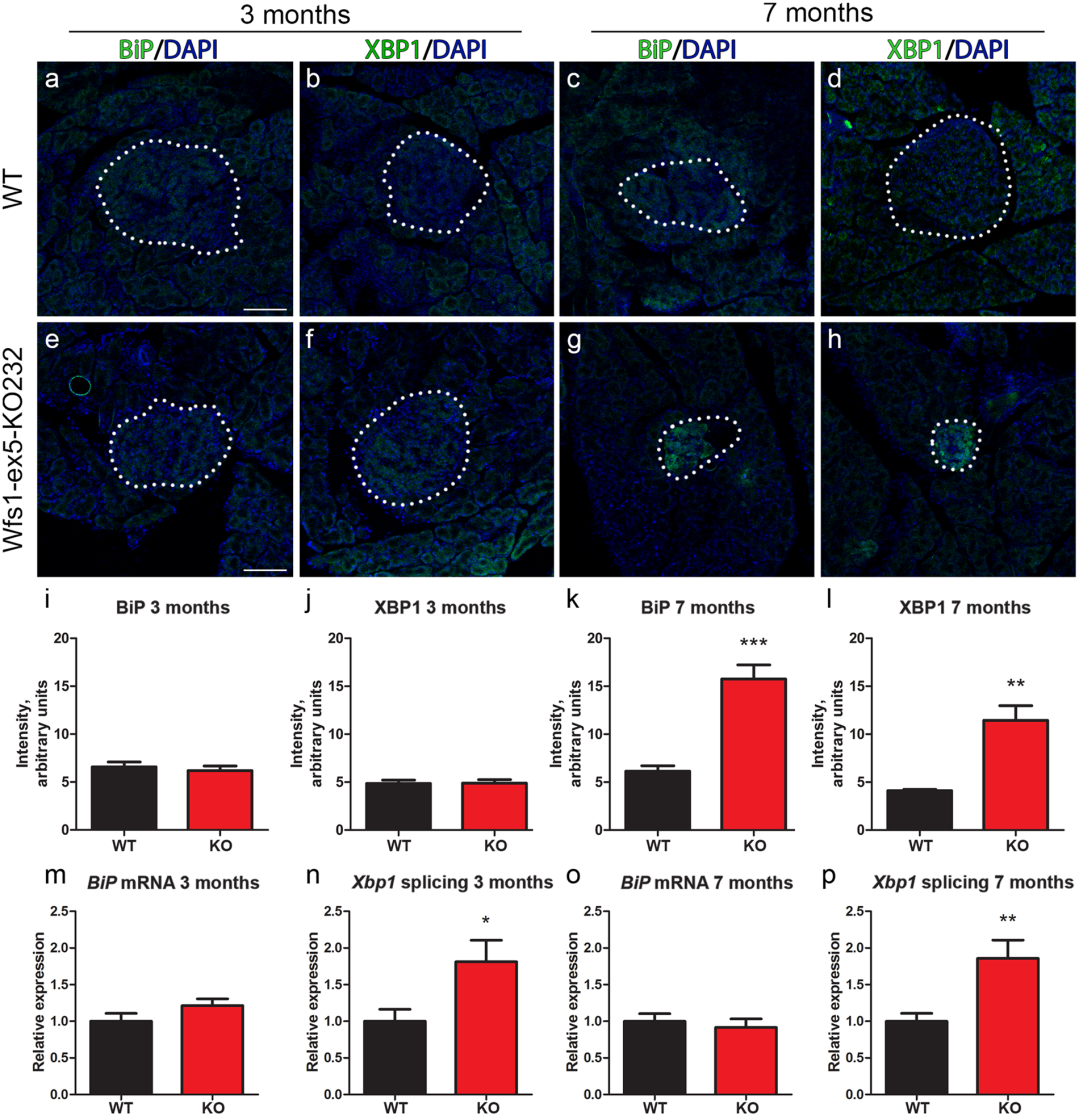
Expression of XBP1 and BiP in islet of Langerhans. Immunofluorescence analysis of endoplasmic reticulum (ER) stress markers **(a**,**e**,**c**,**g)** BiP and **(b**,**f**,**d**,**h)** XBP1 in islets of Langerhans (dotted lines) from 3- and 7-month-old rats. At 3 months, the expression of **(a)** BiP and **(b)** XBP1 in control rats and **(e**,**f)** Wfs1-ex5-KO232 rats was comparable. By 7 months, the expression of **(c)** BiP and **(d)** XBP1 remained at basal level in WT rats, whereas the expression of these ER stress markers was clearly elevated in **(g**,**h)** the islets of Langerhans of Wfs1-ex5-KO232 rats. **(i – l)** Quantification of signal intensity of ER stress markers. Levels of **(k)** BiP and **(l)** XBP1 were increased in islets of Langerhans from 7-month-old Wfs1-ex5-KO232 rats. **(m**,**n**,**o**,**p)** mRNA analysis of ER stress markers *BiP* and spliced *Xbp1* in lysates of isolated islets of Langerhans. Expression level of *BiP* was not altered in **(m)** 3-month- or **(o)** 7-month-old Wfs1-ex5-KO232 rats compared to expression in wild-type rats. *Xbp1* splicing was increased in **(n)** 3-month- and **(p)** 7-month-old Wfs1-ex5-KO232 rats compared to expression in WT rats. The data were compared using *t*-tests; ***p < 0.001, **p < 0.01, *p < 0.05 between genotypes. The data are presented as the mean ± SEM, n = 4 to 9. Cell nuclei (blue) were counterstained with DAPI. Scalebar: 100 µm.

The expression of ER stress markers XBP1 and BiP was evaluated in Wfs1-ex5-KO232 rat pancreases (Fig. 3). The data revealed an uniform expression of ER stress regulators in pancreases from three-month-old rats (Fig. 3a,b,e,f). By seven months of age, the expression of XBP1 and BiP were at the basal level in WT pancreases (Fig. 3c,d) and in the exocrine portion of pancreases of Wfs1-ex5-KO232 rats (Fig. 3g,h). Despite the reduced size of the endocrine portion of pancreases, the level of ER stress markers was increased in the pancreases of Wfs1-ex5-KO232 rats (Fig. 3g,h). At 7 months, there was a significant increase in the level of BiP (p < 0.001, *t*-test) (Fig. 3k) and XBP1 (p < 0.01, *t*-test) (Fig. 3l). ER stress is measured by mRNA expression of BiP and by Xbp1 splicing, and these were evaluated in isolated Wfs1-ex5-KO232 islets of Langerhans (Fig. 3m–p). There were no differences in BiP mRNA expression between Wfs1-ex5-KO232 and WT rats at 3 or 7 months of age (Fig. 3m,o). Compared to islets from WT rats, Xbp1 splicing was increased by 3 months of age in islets from Wfs1-ex5-KO232 rats (p < 0.05) (Fig. 3n), and this difference was further exacerbated in 7-month-old Wfs1-ex5-KO232 rats (p < 0.01) (Fig. 3p).

### Wfs1-ex5-KO232 rats developed cataract, retinal gliosis and displayed elevated ER stress in the retina

**Figure 4:**
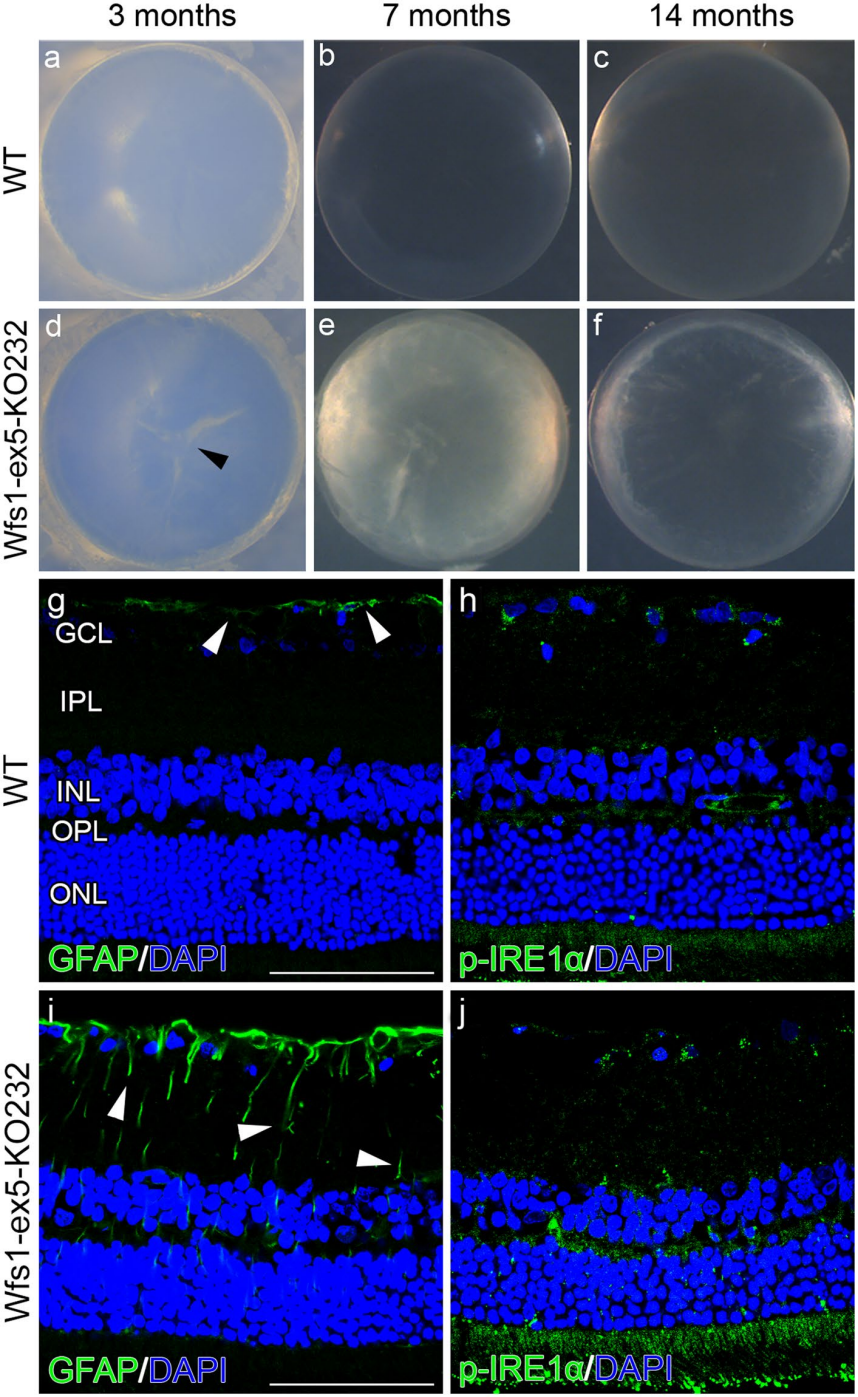
Development of cataracts and the localization and expression of glial fibrillary acidic protein and phospho-IRE1α in retinas. Dissected lenses from (**a–c**) wild-type (WT) and (**d–f**) Wfs1-ex5-KO232 rats. (**d**) 3-month-old Wfs1-ex5-KO232 lenses displayed greater signs of developing cataracts (arrowhead), with a prevalence of 2/5, compared to controls (0/4). (**e**) In 7-month-old knock-out rats, various degrees of cataract progression were seen, with a prevalence of 3/5. (**f**) All observed 14-month-old Wfs1-ex5-KO232 rat lenses (3/3) expressed clear signs of cataracts. Expression and immunolocalization of (**g**,**i**) glial fibrillary acidic protein (GFAP) and the endoplasmic reticulum stress marker (**h**,**j**) phospho-IRE1α in 15-month-old rats. (**g**) Retinas of WT rats showed GFAP expression in astrocytes at the ganglion cell layer (GCL). (**i**) Retinas displayed a clear upregulation of GFAP in Müller glial processes (arrowhead). Phospho-IRE1α in (**h**) WT rats showed a uniform immunoreactivity over the entire retina, whereas in (**j**) Wfs1-ex5-KO232 retina, a robust increase was observed. (**g–j**) Nuclei were counterstained with DAPI (blue). ONL, outer nuclear layer; OPL, outer plexiform layer; INL, inner nuclear layer; IPL, inner plexiform layer. Scalebar: 100 µm.

Lenses from 3-month-old Wfs1-ex5-KO232 rats displayed signs of developing cataracts (Fig. 4d), with the prevalence of 2/5. Severe signs of cataracts were seen in 7-month-old knock-out rats (Fig. 4e; prevalence, 3/5), and seemed to develop independently from diabetes. All lenses from 14-month-old Wfs1-ex5-KO232 rats showed clear signs of cataracts (Fig. 4f; prevalence, 3/3). However, WT rat lenses showed no evidence of cataract regardless of age. (Fig. 4a, 0/4; 4b, 0/5; 4c, 0/4).

Immunolocalization of glial fibrillary acidic protein (GFAP) in WT rats was limited to astrocytes on the inner retinal surface, with no or only slight staining in deeper layers (Fig. 4g). However, the expression of GFAP in Wfs1-ex5-KO232 rats was visibly increased in astrocytes and was upregulated in Müller glial cells (Fig. 4i), indicating Müller cell gliosis.

To determine whether the overall development of Wfs1-ex5-KO232 rats is accompanied with increases in ER stress marker expression in the retina, we evaluated the expression of phospho-IRE1α (Fig. 4h,j). In control rats, the expression of phospho-IRE1α showed uniform immunoreactivity throughout the retina (Fig. 4h), whereas a clear increase in phospho-IRE1α expression was seen in the retinas of 14-month-old Wfs1-ex5-KO232 rats (Fig. 4j).

### Pathological changes in optic nerves of Wfs1-ex5-KO232 rats

**Figure 5:**
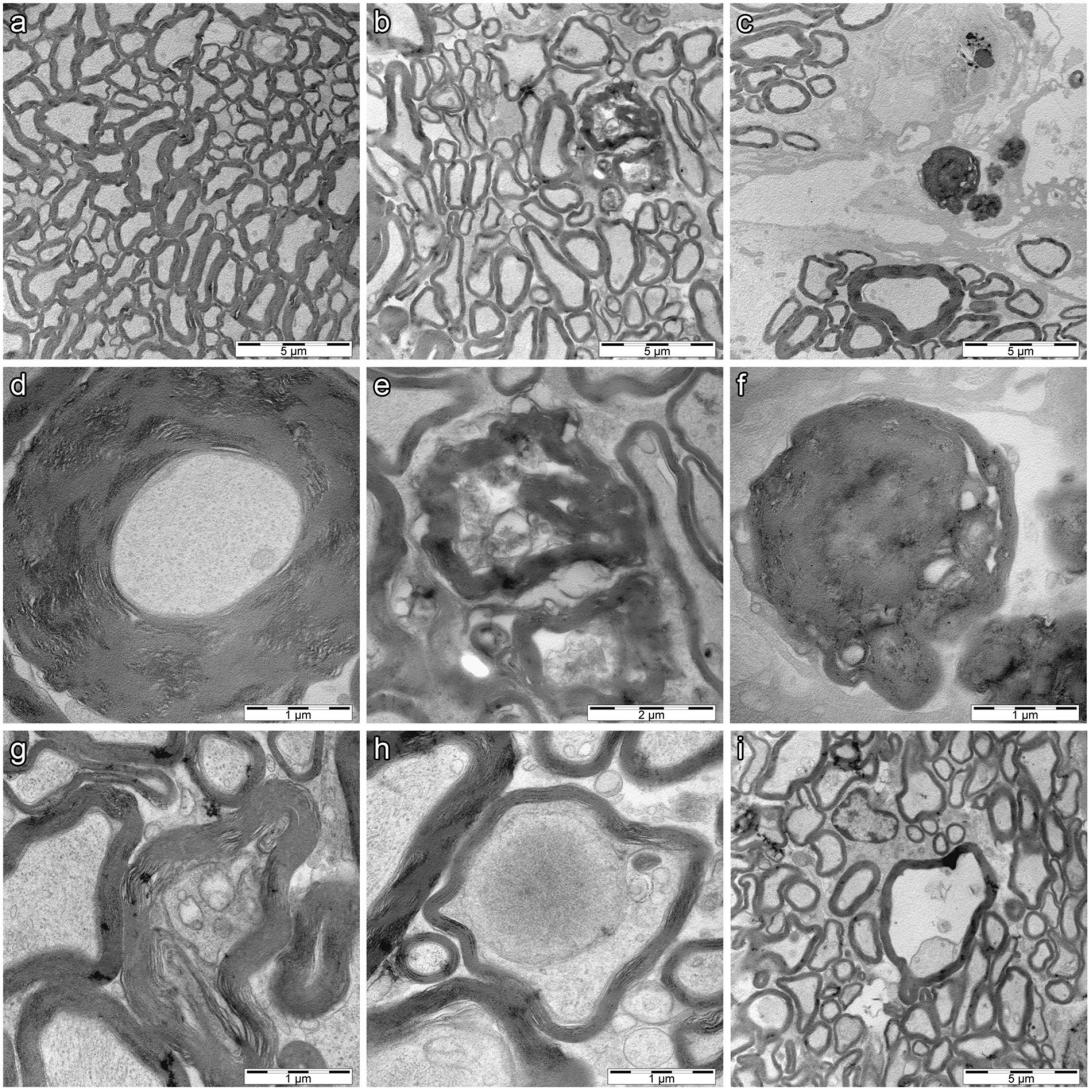
Ultrastructure of the optic nerve in 15-month-old wild-type and Wfs1-ex5-KO232 rats. Low-magnification image shows axons in **(a)** wild-type (WT) and **(b**,**c)** Wfs1-ex5-KO232 rats. **(a)** Axons are tightly packed and show orderly appearance in WT rats. **(b)** Axons are more disorganized and space between axons is increased in Wfs1-ex5-KO232 rats. Axons in knock-out rats show **(d)** hypermyelination as indicated by the thickened myelin sheath. (**e**,**f)** Higher magnification images from (**b**) and (**c**) show necrotic fibres in Wfs1-ex5-KO232 rats. **(g)** Axonal compression and (**h**,**i**) demyelination, with extensive vacuolization is clearly evident in the Wfs1-ex5-KO232 nerve. Images are representative, 4 animals per genotype were analysed.

Transmission electron microscopy of optic nerves revealed clear degenerative processes in Wfs1-ex5-KO232 rats. The number of axons was decreased by approximately 20–25% compared to the number in WT optic nerves. Electron microscopy revealed the presence of hypermyelinization and necrotic axons in all analysed male mutant Wfs1-ex5-KO232 rats at 14–15 months of age (n = 5) (Fig. 5b–i). None of the age-matched WT controls displayed such disturbances (n = 4).

### Optic nerve volume was reduced in Wfs1-ex5-KO232 rats

**Figure 6:**
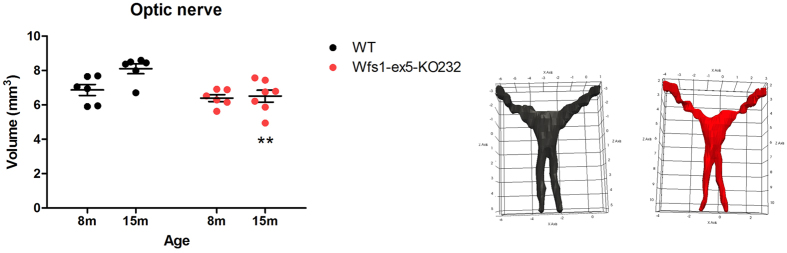
*In vivo* T2-weighted RARE MR imaging of the optic nerve. Wild-type (WT) and Wfs1-ex5-KO232 rats were anaesthetized and imaged at 8 months (WT, n = 6; Wfs1-ex5-KO232, n = 6) and 15 months (WT, n = 6; Wfs1-ex5-KO232, n = 7). The optic nerve was segmented manually by an observer blinded to the genotypes of the rats using IDK-SNAP (V 3.6.0). Left: By 15 months, Wfs1-ex5-KO232 rats showed optic nerve atrophy (optic nerve = optic nerve, chiasm and tract); Right: 3D renderings of the optic nerve (WT, black; Wfs1-ex5-KO232, red) generated using IDK-SNAP and Paraview (V 4.4.0). The data were analysed using a completely randomized ANOVA followed by Fisher’s LSD post hoc tests (see text for details); **p < 0.01 between genotypes. The data are presented as the mean ± SEM.

*In vivo* magnetic resonance (MR) imaging was performed on male WT and Wfs1-ex5-KO232 littermates at 8–15 months of age using a 9.4T preclinical MRI (Fig. 6). At 8 months, no change was detected in Wfs1-ex5-KO232 rats compared with WT littermates. However, by 15 months, optic nerve volume was reduced in Wfs1-ex5-KO232 rats versus that of WT littermates (Fig. 6). WT rats showed an increase in volume over time, whereas no change was detected in Wfs1-ex5-KO232 rats (Fig. 6).

### Wfs1 protein was expressed in the brainstem

**Figure 7:**
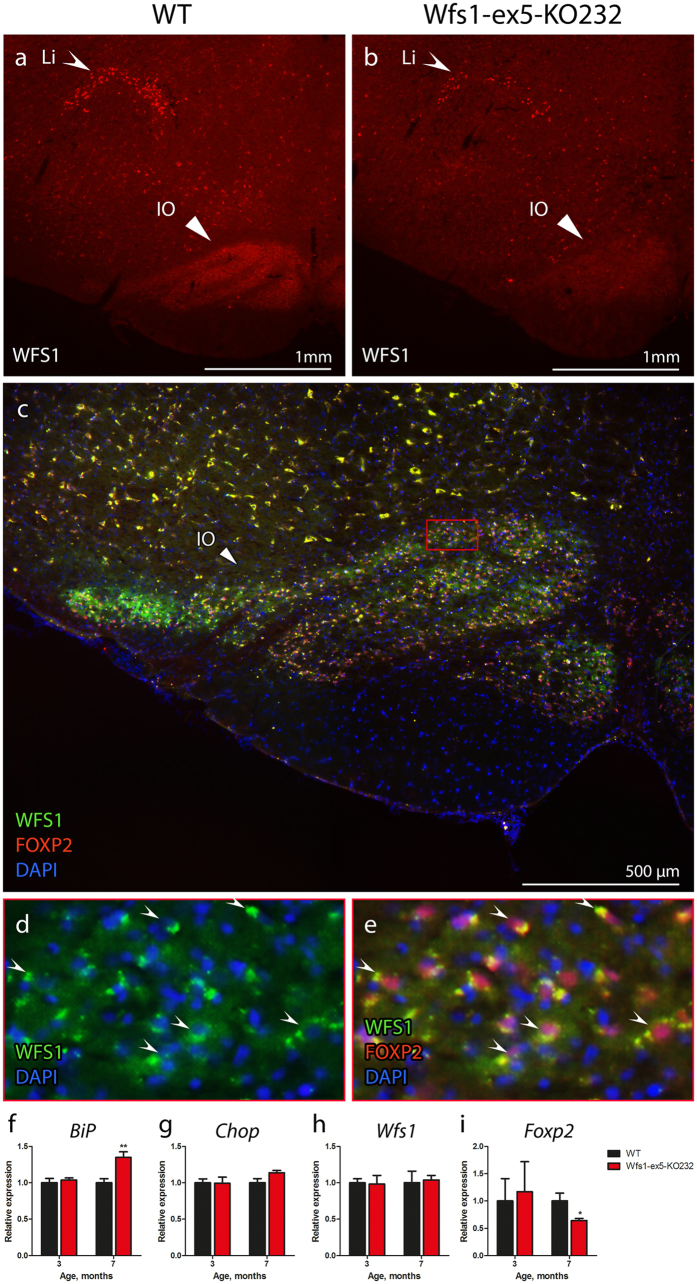
Localization of WFS1 protein in the medullas. Coronal sections of the medullas at the level of inferior olive (bregma −12.60 mm) were stained with anti-WFS1 antibody. WFS1 staining was localized mainly to the linear nucleus (LI) and inferior olive (IO). WFS1 staining was less intense in the medulla of 15-month-old **(b)** Wfs1-ex5-KO232 rats than in that of **(a)** wild-type (WT) rats of the same age. **(c)** WFS1 showed a similar expression pattern with that of the IO neuronal marker FOXP2. **(d)** Higher magnification of the IO shows the cytoplasmic localization of WFS1. **(e)** WFS1 and FOXP2 were expressed in the same set of cells in the IO. **(f)** Real-time PCR analysis of the ER stress marker *BiP* showed an increase in ER stress in the ventral medullas of 7-month-old Wfs1-ex5-KO232 rats but not in those of 3-month-old animals. **(g)** Expression of the ER stress chaperone *Chop* was similar between genotypes at both time points. **(h)** Expression of *Wfs1* mRNA was similar in 3- and 7-month-old Wfs1-ex5-KO232 and WT rats. **(i)** Expression of *Foxp2* was similar in 3-month-old and decreased in 7-month-old Wfs1-ex5-KO232 rats compared to expression in WT animals. The data were compared using two-way ANOVAs followed by Tukey’s HSD tests; *p < 0.05, **p < 0.01 between genotype. The data are presented as the mean ± SEM, n = 8 to 10.

Immunohistochemical analysis of medulla showed localization of WFS1 proteins to the linear nucleus (LI) and inferior olive (IO) (Fig. 7a,b). The intensity of WFS1 staining in 15-month-old Wfs1-ex5-KO232 rats was lower than in WT littermates (Fig. 7a,b), likely indicating the disappearance of WFS1-positive cells in this brain region. Subsequent immunohistological analysis of the IO revealed that WFS1 was expressed exclusively in FOXP2-positive neurons (Fig. 7c,d,e). Quantitative analysis of mRNA expression demonstrated a time-dependent change in *Foxp2* levels between genotypes, namely, at 7 months of age, Wfs1-ex5-KO232 rats showed decreased levels of *Foxp2* expression, although *Wfs1* expression remained unchanged (Fig. 7h,i).

### ER stress was detected in the brainstems of Wfs1-ex5-KO232 rats

Further study of mRNA expression levels demonstrated time-dependent increases in ER stress in the IO of Wfs1-ex5-KO232 rats (Fig. 7f,g). Three-month-old Wfs1-ex5-KO232 rats showed no increase in the ER stress markers *BiP* and *Chop* compared to levels in WT controls (Fig. 7f,g). At 7 months of age, the ER stress marker *BiP* showed increased levels in Wfs1-ex5-KO232 rats compared to those in WT animals (p < 0.01) (Fig. 7f).

### Medullary volume was reduced in Wfs1-ex5-KO232 rats

**Figure 8:**
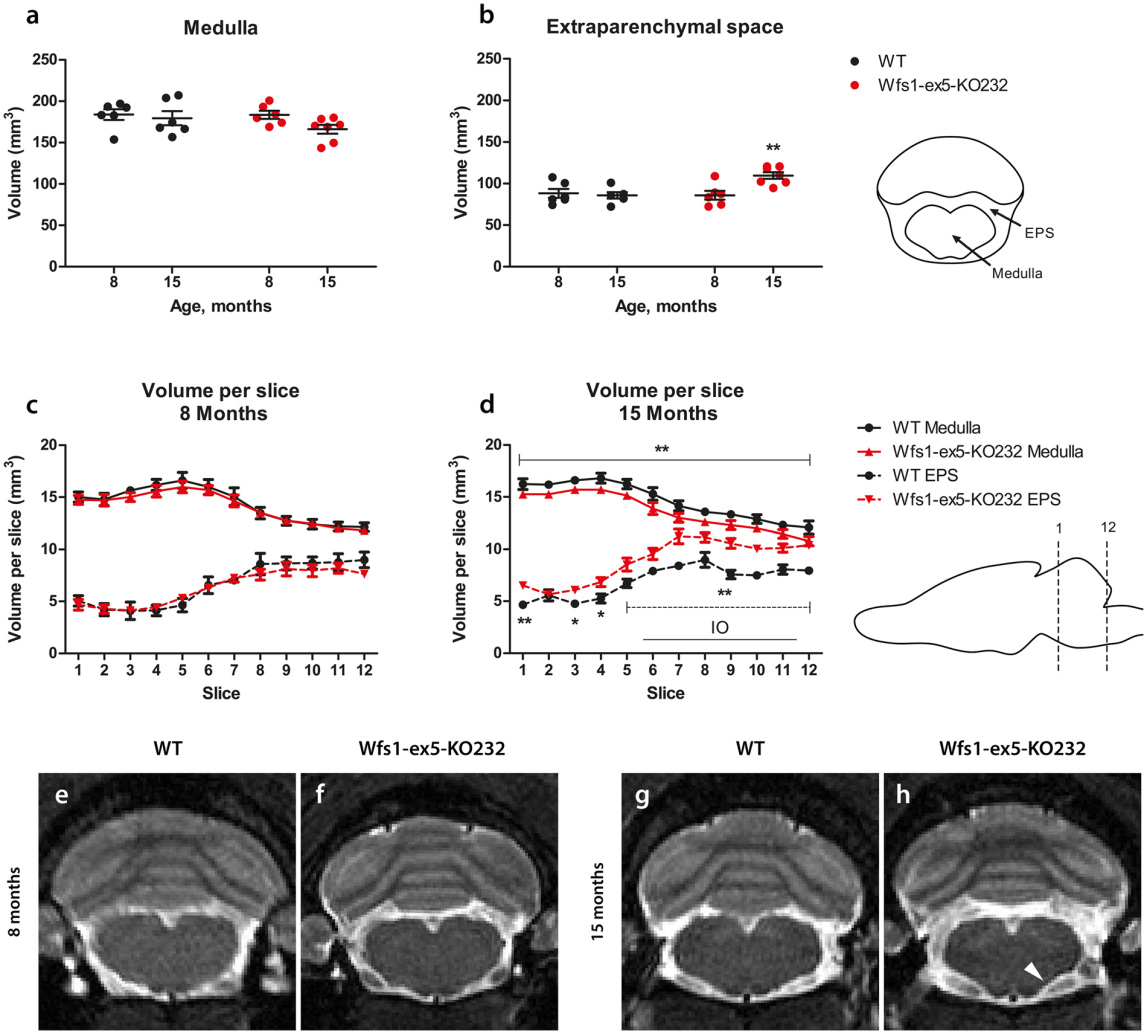
Quantitative MRI analysis of brainstem volume of Wfs1-ex5-KO232 rats. The medulla was manually traced by an observer blinded to the genotypes of the rats from T2 images using ITK-SNAP software. The volumes of the segmented structures were calculated as volume per slice from bregma level −9.48 to −15.48 mm. **(a)** The total volume of the medulla and **(b)** volume of the extraparenchymal space (EPS). Volumes of individual MRI slices from **(c)** 8-month- and **(d)** 15–month-old animals. The volume of the extraparenchymal space is increased at the level of the inferior olive (IO) in Wfs1-ex5-KO232 rats at 15 months. Line shows localization of IO (bregma −12.00 until −14.76 mm). Representative T2-weighted MR images of the medulla of wild-type (WT) and Wfs1-ex5-KO232 rats are taken at the level of the IO (bregma approx. −12.63 mm). **(e**,**f)** There are no significant changes in medullary volume at 8 months of age. At 15 months of age, the ventral surface of the medulla appear more concave (arrowhead) in **(h)** Wfs1-ex5-KO232 rats than in **(g)** WT littermates. **(h)** The area of the extraparenchymal space surrounding the medulla is also increased in Wfs1-ex5-KO232 rats. The data were compared using two-way ANOVAs followed by Fisher’s LSD tests; *p < 0.05, **p < 0.01 between genotypes. The data are presented as the mean ± SEM, n = 6–7.

*In vivo* MRI analysis revealed no difference in medullas (Fig. 8) between Wfs1-ex5-KO232 rats and WT littermates at 8 months of age. The total medullary volume in 15-month-old Wfs1-ex5-KO232 rats was reduced relative to that in WT rats, although the difference was not statistically significant (Fig. 8a and d) (F(1, 143) = 4.08, p = 0.0684, ns (genotype)). A Fisher’s LSD post hoc test revealed that medullary volume in each slice was smaller in 15-month-old Wfs1-ex5-KO232 rats compared to that in WT rats (Fig. 8d). Additionally, the volume of the extraparenchymal space around the medulla was increased in 15-month-old Wfs1-ex5-KO232 rats compared with that of WT littermates (Fig. 8h), quantification of MRI images at the level of the medulla revealed an increase in the total extraparenchymal space in older Wfs1-ex5-KO232 rats (Fig. 8b) (F(1, 21) = 5.57, p < 0.05 (genotype); F(1, 21) = 5.4, p < 0.05 (age); F(1, 21) = 8.003, p < 0.01 (genotype × age)). Moreover, the volume of the extraparenchymal space in each slice was also increased in older Wfs1-ex5-KO232 rats (Fig. 8d) (F(1, 143) = 29.76, p < 0.001 (genotype); F(11, 143) = 32.035, p < 0.0001 (slice); F(11, 143) = 2.305, p < 0.05 (genotype × slice)).

## Discussion

WS is a complex disease and is characterized by diabetes mellitus, diabetes insipidus, optic nerve atrophy, hearing loss, neurodegeneration and a number of other complications^8^. The first symptom to appear in WS patients is usually diabetes mellitus at a median age of 5–6 years, followed by a loss of vision due to optic nerve atrophy^14–16^. WS is often accompanied by neurological and psychiatric symptoms, including anxiety and depression, and a progressive loss of sensory functions, including hearing and taste. The cause of death is usually related to neurodegeneration of the brainstem^7^. There is no cure for WS, and thus, there is a high need for animal models that fully reflect the signs and symptoms of WS in humans.

Zinc-finger technology was used to disrupt the *Wfs1* gene in rats. Targeting exon 5 of the rat *Wfs1* gene was predicted to have minimal off-target effects; therefore, exon 5 of the rat *Wfs1* gene was chosen for mutation. Furthermore, the sequence of the WFS1 protein arising from exon 5 of the rat *Wfs1* gene is identical in rats, mice and humans. Such a high degree of conservation probably indicates the functional importance of the corresponding protein region. Three different rat lines with successful mutations in exon 5 of the rat *Wfs1* gene were created, and mutations were confirmed by sequencing genomic DNA and cDNA. All of these lines showed similar glucose intolerance at 7 months of age (Supplementary material S2). The similar phenotypes of these lines indicate that the mutations were most likely not caused by an off-target effect. Furthermore, we used an outbred rat line, thus further minimizing the possibility of effects due to genetic drift. One line was chosen for further characterization; however, heterozygous animals from all three lines are being maintained at our laboratory for possible future studies. This study was performed on male Wfs1-ex5-KO232 rats, which develop diabetes mellitus at approximately 12 months of age. Female Wfs1-ex5-KO232 rats develop diabetes mellitus later, at approximately at 14–16 months of age (data not shown). Such a difference between sexes might be caused by a protective effect of oestrogen against diabetes, which was previously suggested in Spontaneous Diabetic Torii rats^17^. It would be interesting to evaluate the development of WS in ovariectomized female Wfs1 mutant rats to identify whether oestrogen indeed offers any protection against development of WS in rats.

Several Wfs1 mutant mice lines have been created to model WS. However, Wfs1 mutant mice do not develop diabetes to the same extent as human patients with WS. Wfs1 mutant mice, created in our laboratory, showed glucose intolerance and significantly lower levels of insulin but only slightly elevated blood glucose levels at 6 and 8 months^12, 13^. Nevertheless, Wfs1 mutant mice do develop glycosuria, which may provide an alternative route for the clearance of glucose and explain the apparent lack of profound hyperglycaemia in these animals^12^. The Wfs1-ex5-KO232 rats display glucose intolerance that is steadily exacerbated with age (Fig. 2g–l) and is accompanied by a reduction of beta cell mass (Fig. 2p,q,r) suggesting that a deficit in insulin release underlies their glucose intolerance. Indeed, glucose-stimulated insulin release is diminished in Wfs1-ex5-KO232 rats (Fig. 2l), whereas the sensitivity to insulin remains similar in Wfs1 mutant and WT rats regardless of age (Fig. 2e,f). This is in good agreement with the mouse model, as Wfs1 KO mice also showed no change in insulin sensitivity but did show a deficit in insulin secretion^12, 13^.

Interestingly, 1-month-old rats of either genotype did not show any considerable increase in blood glucose levels during glucose tolerance tests. This might be due to the relatively late time point at which the first blood sample was taken (30 min) as glucose utilization might be faster in young and growing rats. Starting from approximately 10 months of age, Wfs1-ex5-KO232 rats display glycosuria and start to lose body weight (Fig. 2a,c). Thereafter, an increasing number of Wfs1-ex5-KO232 rats develop hyperglycaemia between 11–12 months of age (Fig. 2b). By 13 months of age, all Wfs1-ex5-KO232 male rats in our study displayed fasting hyperglycaemia. At this time point, mutant rats show clear signs of diabetes mellitus. To the best of our knowledge, none of the older Wfs1 mutant mice have progressed to such a stage of diabetes in our laboratory or elsewhere, and thus, the development of diabetes is more severe in the mutant rats than in the genetically modified mice. An increase in the severity of the phenotype of mutant rats compared with that of mice was also demonstrated in the recently created Mecp2 knock-out rat, a model of Rett syndrome^18^. Similar to the Wfs1-KO mice in our laboratory, Wfs1-ex5-KO232 rats also display reduced body weight (Fig. 2a)^12, 13, 19, 20^. Nevertheless, there is a difference in the dynamics of body weight loss between mouse and rat Wfs1 mutants. Wfs1 mutant rats show a slightly reduced body weight from 3 to 10 months when compared with WT animals, whereas male Wfs1-KO mice in our laboratory stop growing at 8 weeks, and the body weight of male Wfs1-KO mice appears to be constant after that age^19^.

Human WS patients develop diabetes mellitus in childhood at approximately 5–6 years of age; this is followed by optic nerve atrophy at approximately 10 years of age. Diabetes mellitus in male Wfs1-ex5-KO232 rats appears at approximately 1 year of age; at this age, the rat is an adult. This apparent discrepancy between WS onset in rats and human patients (adulthood vs childhood, respectively) can be explained by the degenerative nature of WS. The loss of beta cells and the accompanying development of diabetes mellitus require 5–6 years in human WS patients and only 1 year in Wfs1-ex5-KO232 rats.

Degeneration of the brainstem is a common feature of WS^16, 21^. Immunohistological analysis of the brainstem showed that WFS1 is expressed in the LI and IO within the medulla of the rat (Fig. 7). Moreover, WFS1 staining is decreased in the IO of Wfs1-ex5-KO232 rats at 15 months of age (Fig. 7b). Localization of WFS1 in the IO of the rat is similar to the expression pattern of WFS1 in the mouse brainstem, where the lateral superior olive and superior para-olivary nucleus appear as WFS1-positive^11^. T2-weighted MRI analysis at the level of the IO revealed a decrease in medullary volume and an increase in the volume of the extraparenchymal space in Wfs1-ex5-KO232 rats compared with those of WT animals at 15 months of age (Fig. 8). MR imaging has also shown a loss of hindbrain volume in WS patients^22^. Degeneration of the medulla is well recognized feature of WS^3, 16^, and degeneration of IO has also been reported in human WS patients^21^. Thus, the pattern of brainstem neurodegeneration in Wfs1-ex5-KO232 rats show similarities to the neurodegeneration observed in human WS patients.

Transcription factor FOXP2 is expressed in excitatory neurons of the IO that project to the cerebellum^23^. We therefore asked whether WFS1 is expressed in the same neurons. Staining with anti-WFS1 and anti-FOXP2 antibodies revealed that WFS1 seems to be localized exclusively in FOXP2-expressing neurons of the IO in rats (Fig. 7). Interestingly, the *Foxp2* expression level in the ventral medulla is reduced in 7-month-old Wfs1-ex5-KO232 rats. At the same time there is an increase in ER stress marker expression in this brain region (Fig. 7). Reduction of *Foxp2* expression at this age indicates that *Foxp2* expression is probably negatively regulated by ER stress; alternatively, it may indicate a decrease in the number of FOXP2-positive cells. Further study is needed to evaluate the effect of *Wfs1* mutation on the number and function of the FOXP2-positive neurons in the IO.

Eventual blindness resulting from bilateral optic atrophy is one of the diagnostic features of WS^3, 24^. Here, we examined optic nerve volume in 2 separate groups of litter-matched rats (8 and 15 months old). No difference was observed between the Wfs1-ex5-KO232 and WT rats at 8 months. In the older group, the optic nerve volume was reduced in the Wfs1-ex5-KO232 rats compared to WT littermates of the same age (Fig. 6). Similar reductions in optic nerve volume were noted in Wfs1-KO mice^25^. In this study, the optic nerve volume appeared to increase in WT rats over time. This might be explained by the fact that different batches of animals were used for each age group; however, mutant animals were always compared to WT littermates of the same age. Electron microscopic analysis of the optic nerves of 15-month-old rats revealed disturbed myelin structure (Fig. 5), indicative of ongoing optic nerve degeneration. Furthermore, there were signs of retinal gliosis (Fig. 4) in the retinas of 15-month-old Wfs1-ex5-KO232 rats.

It is not known whether retinal gliosis is a direct result of WFS1 dysfunction in the retina or is caused by the hyperglycaemia that is seen in older mutant animals. Levels of ER stress markers were also increased in the retinas of Wfs1-ex5-KO232 rats, in agreement with reported increases of ER stress in the retinas of Wfs1-KO mice^26^. Increases in the expression of pIRE1α in the inner plexiform layer of Wfs1-ex5-KO232 rats can be attributed to increased ER stress in ganglion cells as the expression of WFS1 was found in these cells^26^. Recent cross sectional studies in a young population of WS patients also revealed almost fully penetrant deficits in visual acuity and colour vision^27^ and an association of retinal nerve fibre layer thinning with disease progression^28^. Importantly, optic nerve atrophy was readily observable *in vivo* in our rat model of WS.

Myelin degeneration during the progression of WS is reported as a key feature that accompanies neurodegeneration in WS patients^29^. Alterations of myelinization are therefore strongly correlated with the severity of WS symptoms in human patients^29^. The alterations in myelin structure that were observed in the optic nerves of older Wfs1-ex5-KO232 rats confirm the link between myelin disorganization and the progression of WS symptoms. Further study is needed to investigate alterations in myelin structure in tissues other than the optic nerve of Wfs1 mutant rats.

Cataracts have been suggested as a phenotypic marker of WS^30^; therefore, we evaluated the incidence of cataracts in Wfs1-ex5-KO232 rats. The first signs of cataracts are present in Wfs1-ex5-KO232 rats at 3 months of age (Fig. 4d), and the incidence of cataracts increased during ageing (Fig. 4e,f). None of the WT littermate rats at our laboratory displayed cataracts at any age. The presence of cataracts in Wfs1-deficient rats further supports that these rats developed WS.

In this study, we measured ER stress markers in the retina, pancreas and medulla. Changes in *BiP* mRNA were not observed. In beta cells, BiP protein expression was increased in 7-month-old Wfs1-ex5-KO232 rats but not in 3-month-old rats (Fig. 3i,k). This was probably due to different expression dynamics of ER stress markers on the mRNA and protein levels. *Xbp1* splicing showed an increase at 3 months of age (Fig. 3m), before the increase in total XBP1 protein level was apparent. By 7 months, both XBP1 expression and *Xbp1* mRNA splicing had increased in Wfs1-ex5-KO232 rats (Fig. 3l,p), indicating ER stress in the remaining islet cells. Similar to effects in beta cells, ER stress was increased in the medullas of Wfs1-ex5-KO232 rats long before diminished medullary volumes were observed (Figs 7, 8). Together, these results demonstrate that ER stress precedes both decreases in brainstem volume and cellular loss in the pancreas in Wfs1-mutant rats.

There are some unexpected observations that we have not quantified but that nevertheless might have an impact in further studies. During the MR imaging procedure, older Wfs1-ex5-KO232 rats required lower isoflurane concentrations to maintain a normal respiration rate. This might indicate a reduction in the activity of the respiratory centre due to the degeneration of the brainstem in these rats. Interestingly, approximately a quarter of the older Wfs1-ex5-KO232 rats required tooth trimming, whereas none of the WT rats required this treatment. WS patients are reported to have dysphagia^7, 14^, and reduced tooth wear might be an indicator of a similar problem in rats. Therefore, an in-depth study is required to evaluate brainstem function in Wfs1-ex5-KO232 rats.

WS has a wide range of symptoms and complications; in this study, we have characterized only some of the most critical features of WS in Wfs1-ex5-KO232 rats. The diagnosis of WS requires the presence of two core symptoms: diabetes mellitus and optic nerve atrophy^31^. The phenotype of Wfs1-ex5-KO232 rats indicates that it has these core symptoms of WS, including progressive insulin-dependent diabetes mellitus, retinal gliosis and cataracts, as well as displaying degeneration of the medulla and optic nerve. Therefore, we present a novel rat model of WS. The severity of the phenotype in this Wfs1-mutant rat more closely mimics the human condition than analogous mouse models. In conclusion, Wfs1-ex5-KO232 rats are a valuable new model to study the molecular mechanism and treatment options for WS. Wfs1 mutant rats can also be used for translational research connected to other ER stress-related disorders, including diabetes and neurodegeneration.

## Materials and Methods

### Animals

Breeding and genotyping were performed at the Laboratory Animal Centre, University of Tartu. For this study, 1 to 16-month-old male homozygous Wfs1-ex5-KO232 Wfs1-deficient (*Wfs1* exon 5 KO) and WT littermate control rats were used. Animals were housed in cages in groups of 3 to 4 animals per cage under a 12 h light/dark cycle (lights on at 7 am). Rats had unlimited access to food and water except during testing. Sniff universal mouse and rat maintenance diet (Sniff cat# V1534) and reverse osmosis-purified water were used. Experiments were performed between 9 am and 5 pm. Permission for this study was given by the Estonian National Board of Animal Experiments (No. 54, 23th of February 2015) in accordance with the European Communities Directive of September 2010 (2010/63/EU).

### ZFN constructs and mRNA

Rat *Wfs1* exon 5-specific zinc-finger nucleases (ZNFs) and microinjection-ready mRNA were obtained from Sigma-Aldrich (CSTZFN-1KT lot: 02091202 MN). The Rat *Wfs1* exon 5 DNA sequence and ZFN binding and cutting sites are described in Fig. 1a.

### Generation of the Wfs1 deficient rat line by ZFN mRNA pronuclear microinjection

Sprague-Dawley rats (Crl: CD(SD) rats CD® IGS, Charles River Laboratories) were housed in standard cages and maintained on a 12 h light/dark cycle with *ad libitum* access to food and water. 4- to 5-week-old embryo donors were superovulated by injection with 20 units of pregnant mare serum gonadotropin (PMSG Sigma Cat. no. G-4877), and 48 h later, just before mating, rats were injected with 50 units of human chorionic gonadotropin (hCG Sigma Cat. no. CG-5). Fertilized egg cells were harvested a day later in M2 medium (Sigma Cat. No. M7167), and cells where incubated in KSOM medium (Specialty Media, Cat. #MR 121-D). For embryo manipulations, ZFN mRNA was injected into the pronucleus of fertilized eggs. The final concentration of each ZFN mRNA was 2.5 ng/μl. For the synchronization of female recipients, rats were injected with 40 μg of LH-Rh 72 h before mating them with vasectomized males. Female rats in the proestrus phase were mated with vasectomized studs at day 0 to induce pseudo-pregnancy. On the following day (day 1), the mated females where inspected for copulatory plugs. Thereafter, microinjected egg cells were transferred to the oviduct of pseudopregnant Sprague-Dawley recipients (max of 40 embryos per female).

### Mutation detection assay

PCR analysis was conducted to amplify a 333-bp region surrounding the target site. The resulting PCR products were pyrosequenced. The primer set for the PCR genotyping analysis was as follows: rwfs_zf_genoR1 (5′-AAGAGTGGGTATGGTGCTGG-3′) and rwfs_zf_genoF1 (5′-AGAAGTGGCTACCCAGGGAT-3′). In the mutation detection assay, we found that founder line 232 had 2 bands, indicating a possible large deletion in exon 5 of the *Wfs1* gene. Both of the PCR products were extracted from the agarose gel and analysed via DNA pyrosequencing (Fig. 1b). Pyrosequencing primers were as follows: rwfs_zf_genoR1 (5′-AAGAGTGGGTATGGTGCTGG-3′) and rwfs_zf_genoF1 (5′-AGAAGTGGCTACCCAGGGAT-3′).

### cDNA analysis for verification of the Wfs1 mutation

Total RNA was extracted individually from the hearts of WT and WFS1ex5-KO232 rats using TRIzol® reagent (Invitrogen, USA) according to the manufacturer’s protocol. First-strand cDNA was synthesized using poly(T)18 oligonucleotides and SuperScript™ III Reverse Transcriptase (Invitrogen, USA). Primers for cDNA genotyping and pyrosequencing were as follows: Wfsex4 F (5′-TCACTTCTGAGAATGAGGCCG-3′) and Wfsex7 R (5′-ATGAGGGCGTTGATGTGATGG-3′).

### Intraperitoneal glucose tolerance tests

Animals were deprived of food for 3 h before and during the experiment; water was available throughout the experiment. Glucose (Sigma-Aldrich) was dissolved in 0.9% saline solution (20% w/vol) and administered intraperitoneally at a dose of 2 g/kg of body weight. Blood glucose levels were measured at the indicated time points from the tail vein using a hand-held glucometer (Accu-Check Go, Roche, Mannheim, Germany). Blood samples were drawn from the tail vein immediately before and 30 min after glucose administration to measure blood insulin levels.

### Insulin measurements

Serum was separated using standard procedures, and serum insulin levels were measured using an ultra-sensitive rat insulin ELISA kit (CrystalChem cat# 90060) according to the manufacturer’s instructions.

### Insulin tolerance tests

Insulin tolerance tests were performed similarly to the glucose tolerance tests except that human insulin (1 unit/kg s.c., Lantus Solostar) was administered instead of glucose. The incidence of hyperglycaemia in Wfs1-ex5-KO232 rats increases with time during from 11–12 months of age; therefore, insulin tolerance tests were performed at 11 months of age on animals that had not yet developed hyperglycaemia.

### Immunohistochemistry

Rats were anaesthetized with an intraperitoneal injection of ketamine (100 mg/kg) and dexmedetomidine (20 mg/kg) and were then perfused transcardially with 4% paraformaldehyde (PFA, Sigma-Aldrich) in 0.1 M phosphate buffer (PB, pH 7.4). Tissues of interest (pancreas, medulla and retinas) were dissected and further fixed in the same fixative overnight at 4 °C. Lenses were removed immediately after perfusion and imaged. Tissues were cryoprotected in 30% sucrose (AppliChem)/0.1 M PB solution until they sank and were then frozen at -80 °C until further use. Sections (40 µm thick) were cut using a cryomicrotome (Microm HM-560) and collected on Superfrost Polysine Slides (Thermo Scientific). After washing with phosphate buffered saline (PBS) for 10 min, sections were permeabilized with 0.2% Triton X-100 (Naxo, Tartu, Estonia)/PBS solution for 40 min. Sections were further incubated in a blocking solution containing 5% donkey serum/1% bovine serum albumin (BSA, Sigma-Aldrich)/PBS for 2 h at room temperature. Primary and secondary antibodies were diluted in 0.1% Tween-20/1% BSA/PBS. Sections were incubated with primary antibodies for 12 h at 4 °C and were then washed with PBS for 1 h. Sections were incubated with the appropriate secondary antibody at room temperature for 2 h. After subsequent washes with PBS (1 h), cell nuclei were counterstained with DAPI (4′,6-diamidino-2-phenylindole, Sigma-Aldrich) at a 1:2000 dilution in 0.1% Tween-20/PBS for 15 min and further washed with PBS. Sections were mounted in Vectashield mounting medium (Vector Laboratories Inc.) and covered with a 0.17-mm coverslip (Deltalab). Images were taken with an Olympus FV-1000 (Olympus) confocal microscope or Olympus BX51 Fluorescence Microscope and annotated with Adobe Photoshop CC (Adobe Systems Incorporated).

Primary antibodies and their dilutions were as follows: mouse anti-insulin (1:500, Santa Cruz Biotechnology Cat# sc-9168, RRID:AB_2126540), goat anti-WFS1 (1:200, Santa Cruz Biotechnology Cat# sc-47936, RRID:AB_2216169), rabbit anti-phospho-IRE1α (1:750, Novus Cat# NB100–2323, RRID:AB_10145203), rabbit anti-XPB1 (1:500, Abcam Cat# ab 37151, RRID:AB_778942), rabbit anti-BiP (1:200, Cell Signalling Technology Cat# 3177 P, RRID:AB_10828008), rabbit anti-GFAP (1:1000, Synaptic Systems Cat# 173 002, RRID:AB_887720), goat anti-FOXP2 (1:400, Everest Biotech, Cat# EB05226, Lot# 160409), and rabbit anti-WFS1 (1:400) as previously described^11^. Secondary antibodies and their dilutions were as follows: FITC AffiniPure donkey anti-rabbit (1:1000, Jackson ImmunoResearch Lab., 711–095–152, RRID:AB_2315776), Alexa Fluor® 488 AffiniPure donkey anti-mouse (1:1000, Jackson ImmunoResearch Lab., 715-545-150, RRID:AB_2340846), TRITC AffiniPure donkey anti-mouse (1:1000, Jackson ImmunoResearch Lab., 715-025-150, RRID:AB_2340766), and Rhodamine Red-X-AffiniPure rabbit anti-goat (1:1000, Jackson ImmunoResearch Labs Cat# 305-297-003, RRID:AB_2339496).

The WFS1 protein in Wfs1-ex5-KO232 rats lacks 27 amino acids (aa 212-238). The commercial anti-WFS1 antibody is targeted to the C-terminus of the WFS1 protein. Therefore, the mutated WFS1 protein in Wfs1-ex5-KO232 rats is still recognized by this antibody.

Histological analyses were always performed using material from 3 to 7 animals per genotype from each age group. Representative images for each group are shown. Fluorescence intensity measurements of ER stress markers were performed using ImageJ software (NIH, Bethesda, MD, USA). Specifically, 8-bit images from optical sections approximately 5 µm thick were converted to greyscale, and intensity values were measured as the mean pixel value in the islets of Langerhans.

### Determination of beta cell mass

Beta cell mass was estimated as previously described^32^. Briefly, rats were perfused, their pancreases were dissected and excess fat was removed. The weight of each pancreas was recorded, and the tissue was processed for histological analysis as described above. 40-micrometre-thick serial sections were cut at intervals of 240 micrometres, stained with anti-insulin antibody and photographed using a Leica SCN 400 slide scanner at 10x magnification. Obtained images were analysed using ImageJ software. Beta cell mass for each animal was estimated by dividing the total islet area by the total pancreas area, and the obtained relative islet area was multiplied by the weight of the pancreas to estimate beta cell mass.

### Electron microscopy

Optic nerves were carefully removed and immersion-fixed in 4% PFA/2.5% glutaraldehyde (GA, AppliChem) solution in 0.1 M PB for 2 h at room temperature. After washing in 90 mM sodium-cacodylate buffer (Sigma-Aldrich), nerves were post-fixed in 1% osmium tetra-oxide (Sigma-Aldrich) solution in 90 mM sodium-cacodylate buffer. After washing, tissues were dehydrated and embedded in epoxy resin (medium hardness, TAAB). Ultrathin sections (60–90 nm) were made using a diamond knife (Diatome) mounted on a PowerTome MT-XL RMC (RMC Products, Boeckeler Instruments) ultra-microtome. Sections were contrasted using 2% uranyl acetate in 50% ethanol (w/v) and 0.2% lead citrate in 0.1 M sodium hydroxide (pH 12). Electron microscopic analysis of specimens was performed using a Tecnai 10 transmission electron microscope (FEI, Netherlands). Images were annotated with Adobe Photoshop CC (Adobe Systems Incorporated) software. The counting of ganglion cells and measurements of their axonal cross sectional area were performed on 3 to 5 animals from each age and genotype group. Three randomly obtained images with areas of 1600 square micrometers each were analysed for every animal using ImageJ software.

### Urine chemistry

Urine was collected during the light phase from non-fasted rats. Urine glucose levels were determined using standardized procedures at the United Laboratories of Tartu University Hospital.

### Isolation of islets of Langerhans

Islets of Langerhans were isolated as previously described^33^. In brief, collagenase solution was injected into the common bile duct of euthanized animals; inflated pancreases were collected, and tissues were enzymatically dispersed. Islets of Langerhans were collected by hand from exocrine tissue under a stereo microscope and allowed to recover in RPMI 1640 medium supplemented with 10% foetal bovine serum and penicillin (100 U/mL)/streptomycin (100 μg/mL) over night before RNA isolation.

### RNA isolation, cDNA synthesis and gene expression analyses

Total RNA from ventral medullas was extracted using TRIzol® reagent (Invitrogen, USA) according to the manufacturer’s protocol. RNA from islets of Langerhans was isolated using Direct-zol RNA MiniPrep (Zymo Research) according to the manufacturer’s protocol. First-strand cDNA was synthesized using random hexamers and SuperScript™ III Reverse Transcriptase (Invitrogen, USA). Real-time quantitative PCR using TaqMan Gene Expression Assays (Thermo Fisher Scientific) and Taqman Gene Expression Mastermix (Thermo Fisher Scientific) was used for analysing *Chop* (Rn00492098_g1), *BiP* (Rn00565250_m1), *FoxP2* (Rn01456150_m1) and *Wfs1* (Rn00582735_m1) expression. Relative quantification was performed using the 2^−∆Ct^ method, with *Hprt1* (Rn01527840_m1) as an internal control. *Xbp1* splicing was analysed using rat *Xbp1*-specific PCR as previously described^34^. Integrated density levels were measured using ImageJ software. All gene expression results were normalized to their expression in WT rats.

### *In vivo* magnetic resonance imaging

Rats at 8 months (n = 6, WT; n = 6, KO) and 15 months of age (n = 6, WT; n = 7, KO) were anaesthetized using isoflurane (1.5–2.5% in 1.5 l/min medical oxygen) and placed on a heated animal bed throughout the MR procedure. All scans were performed using a 9.4T Bruker BioSpec 94/20 USR system connected to a 1 H circular polarized transceiver coil and running ParaVision 6.0.1® software (Bruker BioSpin Group, Bruker Corporations, Germany). Respiration and temperature were monitored using a respiration pillow and a rectal probe (SA Instruments Inc., Stony Brook, USA). Respiration rate was maintained at between 35–70 breaths per minute. Two orientation pilot scans were performed in order to establish the position of the animal and identify anatomical landmarks relevant for planning the subsequent scan. The final T2-weighted Turbo RARE sequence was performed using the following parameters: repetition time (TR) 6803 ms, echo time (TE) 33 ms, flip angle 90 degrees, number of averages 5, imaging matrix 320 × 320 × 65, spatial resolution 0.16 × 0.16 × 0.5 mm.

Volumes were segmented manually by an observer blinded to the genotype using ITK-SNAP (V3.6.0)^35^. For the optic nerve + chiasm + tract, segmentation began where the optic nerves emerged through the optic canal foramina and continued until the point where the optic tract was no longer discernable from surrounding parenchyma. For the medulla, segmentation began after the inferior colliculus and continued until reaching the most caudal level of the cerebellum (bregma −9,48 to −15,48 mm)^36^.

### Data analysis

The data are presented as the mean ± SEM and were compared using Student’s *t*-tests or two-way analysis of variance (ANOVA) followed by Tukey post hoc tests. The data were analysed using GraphPad Prism version 5 software (GraphPad Software Inc., San Diego, CA, USA) or Statistica version 8 software (Statistica, Tulsa, OK, USA). MRI data were analysed using GBStat V 8 (Dynamic Microsystems Inc., Silverspring, MD, USA) using repeated or completely randomized ANOVAs, as appropriate, followed by Fisher’s LSD *post hoc* tests. A p value of < 0.05 was considered statistically significant.

## Electronic supplementary material

Supplementary Material
