# Supplementary material for: Wfs1- deficient rats develop primary symptoms of Wolfram syndrome: insulin-dependent diabetes, optic nerve atrophy and medullary degeneration
Source: Sci Rep. 2017 Aug 31;7:10220. doi: 10.1038/s41598-017-09392-x (PMC5579261; doi:10.1038/s41598-017-09392-x)
Supplement: Supplementary file 1 — Supplementary Material [file 41598_2017_9392_MOESM1_ESM.pdf]

Supplementary Material:

Title: Wfs1- deficient rats develop primary symptoms of Wolfram syndrome: insulin-dependent diabetes, optic nerve atrophy and medullary degeneration

Authors: Mario Plaas, Kadri Seppa, Riin Reimets, Toomas Jagomäe, Maarja Toots, Tuuliki Koppel, Tuuli Vallisoo, Mait Nigul, Indrek Heinla, Riho Meier, Allen Kaasik, Andres Piirsoo, Miriam A Hickey, Anton Terasmaa, Eero Vasar

```
WT                  201    LLENVGQVNE QDGGAQPGPV PKSLQK    QRRM LERLVSSESK  NYIALDDFVE
Wfs1-ex5-KO232      201    LLENVGQVNE Q----------------    -------------AK  NYIALDDFVE
Wfs1-ex5-KO266      201    LLENVGQVNE Q----------------    -------------AK  NYIALDDFVE
Wfs1-ex5-INS244     201    LLENVGQVNE QDGGAQPGPV PKSYCMNTIQRRM LERLVSSESK  NYIALDDFVE
```

Figure S1. Predicted WFS1 protein sequence from three different Wfs1 mutant rat lines. Protein sequence is based on cDNA sequence analysis.

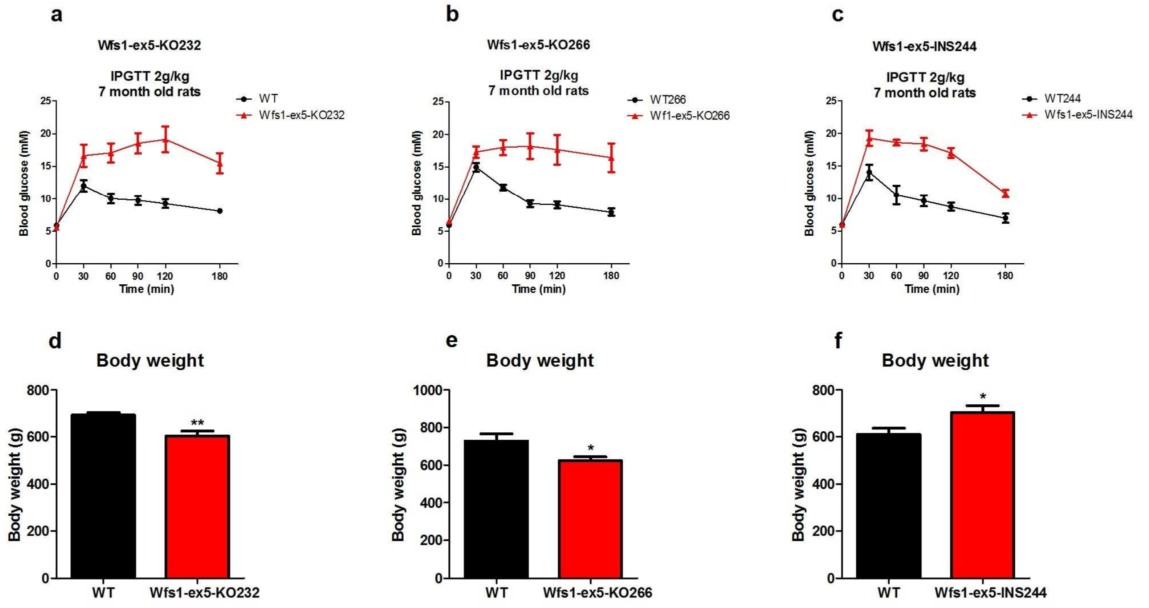

Figure S2. Glucose tolerance and body weight of three different Wfs1 mutant rat-lines at seven months of age (males). (a, b, c) All Wfs1 mutant rats display glucose intolerance at this age. **(d, e)** Wfs1-ex5-KO232 and Wfs1-ex5-KO266 rats are lighter than wild-type littermates. **(f)** Wfs1-ex5-INS244 rats are heavier than littermates. Student’s t-test; $*p < 0.05$, $** < 0.01$ between genotypes. The data are presented as mean ± SEM, n = 4 -7.
